# ^18^F-FLT PET/CT as a Prognostic Imaging Biomarker of Disease-Specific Survival in Patients with Primary Soft-Tissue Sarcoma

**DOI:** 10.2967/jnumed.121.262502

**Published:** 2022-05

**Authors:** Joseph G. Crompton, Wesley R. Armstrong, Mark A. Eckardt, Ameen Seyedroudbari, William D. Tap, Sarah M. Dry, Evan R. Abt, Jeremie Calais, Ken Herrmann, Johannes Czernin, Fritz C. Eilber, Matthias R. Benz

**Affiliations:** 1 Division of Surgical-Oncology, Department of Surgery, UCLA, Los Angeles, California;; 2 Ahmanson Translational Theranostics Division, Department of Molecular and Medical Pharmacology, UCLA, Los Angeles, California;; 3 Department of Surgery, Yale School of Medicine, New Haven, Connecticut;; 4 Department of Medicine, Sarcoma Medical Oncology Service, Memorial Sloan Kettering Cancer Center, New York, New York;; 5 Department of Pathology, UCLA, Los Angeles, California; and; 6 Department of Nuclear Medicine, University of Duisburg–Essen and German Cancer Consortium–University Hospital Essen, Essen, Germany

**Keywords:** ^18^F-FLT PET, sarcoma, imaging biomarker

## Abstract

The purpose of this study was to evaluate ^18^F-FLT PET/CT as an early prognostic imaging biomarker of long-term overall survival and disease-specific survival (DSS) in soft-tissue sarcoma (STS) patients treated with neoadjuvant therapy (NAT) and surgical resection. **Methods:** This was a 10-y follow-up of a previous single-center, single-arm prospective clinical trial. Patients underwent ^18^F-FLT PET/CT before treatment (PET1) and after NAT (PET2). Posttreatment pathology specimens were assessed for tumor necrosis or fibrosis and for Ki-67 and thymidine kinase 1 expression. Maximally selected cutoffs for PET and histopathologic factors were applied. Survival was calculated from the date of subject consent to the date of death or last follow-up. **Results:** The study population consisted of 26 patients who underwent PET1; 16 of the 26 with primary STS underwent PET2. Thirteen deaths occurred during a median follow-up of 104 mo. In the overall cohort, overall survival was longer in patients with a low than a high PET1 tumor SUV_max_ (dichotomized by an SUV_max_ of ≥8.5 vs. <8.5: not yet reached vs. 49.7 mo; *P* = 0.0064). DSS showed a trend toward significance (*P* = 0.096). In a subanalysis of primary STS, DSS was significantly longer in patients with a low PET1 tumor SUV_max_ (dichotomized by an SUV_max_ of ≥8 vs. <8; *P* = 0.034). There were no significant ^18^F-FLT PET response thresholds corresponding to DSS or overall survival after NAT at PET2. **Conclusion:** ^18^F-FLT PET may serve as a prognostic baseline imaging biomarker for DSS in patients with primary STS.

## Visual Abstract

**Figure:**
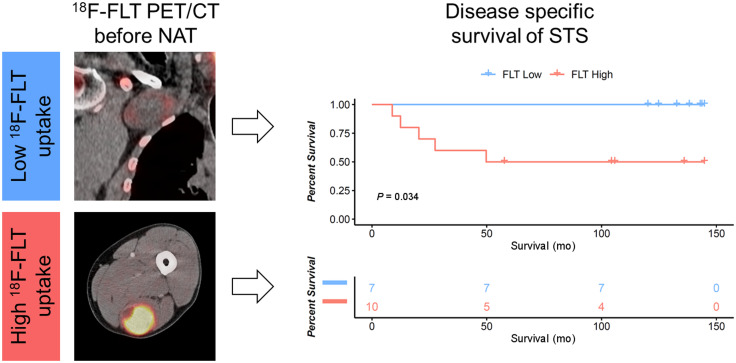

Soft-tissue sarcomas (STSs) comprise approximately 1% of adult cancers (*1*) but constitute a family of more than 50 histotypes (*2*) that present quite differently in biologic characteristics and clinical behavior.

Histologic tumor grading by the French Federation of Cancer Centers Sarcoma Group (FNCLCC) is regarded as the gold standard for prognostication and guides the clinical management of STS patients (*3*). The distinction between low, intermediate, and high grade is determined by 3 parameters: differentiation, mitotic activity, and the extent of tumor necrosis. However, the FNCLCC system has several limitations, including lack of applicability to all sarcoma histotypes, inherent difficulty in reproducibly assessing sarcoma differentiation, and undersampling from core-needle biopsy (*4**,**5*). In addition, the FNCLCC system was developed on untreated tumors. Grading on post–neoadjuvant therapy (NAT) resections in STS is not advised since tumor necrosis cannot be distinguished from NAT-induced necrosis.

Genomic tests might in the future replace or complement current histologic grading in STS (*6*). The complexity index in sarcomas is a prognostic gene expression signature that comprises 67 genes involved in pathways of mitosis control and chromosome segregation (*7*). The complexity index in sarcomas has been identified as a better prognostic factor of metastasis-free survival than the FNCLCC system, irrespective of the STS histotype (*7*).

Proliferative activity–dependent accumulation of 3′-deoxy-3′-fluorothymidine (^18^F-FLT) has been demonstrated for a variety of solid and hematologic neoplasms; however, varying degrees of correlation between ^18^F-FLT uptake and histologic markers of proliferation, such as Ki-67, have been reported (*8**,**9*).

In the current study, we correlated ^18^F-FLT uptake at pre- and post-NAT PET, changes in ^18^F-FLT uptake, and post-NAT histologic variables (percentage tumor necrosis, Ki-67, and thymidine kinase 1 [TK1] expression) with overall survival and disease-specific survival (DSS) in patients previously enrolled in a prospective single-center, single-arm exploratory study. The hypothesis was that ^18^F-FLT PET might be used as a prognostic imaging biomarker of DSS in patients with STS.

## MATERIALS AND METHODS

### Study Design and Patients

Between October 2008 and September 2009, 26 patients with high-grade STS and 1 patient with osteosarcoma were enrolled in a prospective single-center, single-arm exploratory study that investigated the cell proliferation response to NAT as measured by ^18^F-FLT PET/CT (Institutional Review Board [IRB] trial 07-03-110) (*8*). This previous study enrolled adult patients (≥18 y) who were scheduled to undergo NAT before surgical resection of a biopsy-proven sarcoma. Exclusion criteria were unresectable disease, performance status preventing the initiation of NAT, systemic therapy within 6 mo of study participation, a synchronous second malignancy, and the inability to tolerate a PET/CT study. For the purpose of the current study, the patient with osteosarcoma was excluded; therefore, the current study population consisted of 26 patients; 19 of the 26 (73%) had primary disease, and 7 (27%) had recurrent or residual disease. Two of the 19 patients with primary disease had a contemporary history of a secondary malignancy (hepatocellular carcinoma and breast cancer).

All 26 patients underwent ^18^F-FLT PET/CT before initiation of NAT, and 20 patients (77%), after completion of NAT. Six patients did not undergo ^18^F-FLT PET/CT after NAT (PET2): two of these patients exhibited a low SUV_max_ at ^18^F-FLT PET/CT before treatment (PET1) (SUV_max_, 1.7 and 2.0), two had a further diagnostic workup after PET1 that revealed unresectable disease, one had a synchronous secondary malignancy at the time of PET 1 (hepatocellular carcinoma), and one declined to undergo PET2.

The median interval between treatment initiation and PET1 and between PET1 and PET2 was 0.7 wk (interquartile range [IQR], 0.1–1.5 wk) and 11 wk (IQR, 10–16.7 wk), respectively. The patient demographics and clinical characteristics are summarized in Tables 1 and 2.

**TABLE 1.** Clinical and Pathologic Characteristics (*n* = 26)

Characteristic	Data
Median age (y)	63 (range, 26–94)
Sex	
Male	13 (50%)
Female	13 (50%)
Site	
Extremity	12 (46%)
Chest/trunk	8 (31%)
Retroperitoneal/abdominal	6 (23%)
Presentation status	
Primary	17 (65%)
Primary + contemporary history of secondary malignancy	2 (8%)
Recurrent or residual	7 (27%)
Tumor size	
<5 cm	6 (23%)
5–10 cm	13 (50%)
>10 cm	7 (27%)
Histology	
NOS	7 (27%)
MPNST	3 (12%)
Gastrointestinal stromal tumor	3 (12%)
Angiosarcoma	2 (8%)
Leiomyosarcoma	5 (19%)
Fibromyxoid sarcoma	3 (12%)
Pleomorphic liposarcoma	1 (4%)
Dedifferentiated liposarcoma	1 (4%)
Synovial sarcoma	1 (4%)

NOS = sarcoma not otherwise specified; MPNST = malignant peripheral nerve sheath tumor.

Data are number followed by percentage in parentheses, except for age.

**TABLE 2.** Treatment Characteristics (*n* = 26)

Characteristic	Data
NAT	
CTx (including Gleevec)	11 (42%)
CRTx	10 (38%)
RTx	2 (8%)
No neoadjuvant	3 (12%)
Surgery	24 (92%)
Adjuvant therapy	
CTx	11 (42%)
CRTx	3 (12%)
RTx	2 (8%)
No adjuvant therapy	6 (23%)
Incomplete records	4 (15%)
Recurrent therapy	
CTx	3 (12%)
Surgery	3 (12%)
Surgery + CTx	2 (8%)
Surgery + RTx	1 (4%)
Surgery + CRTx	1 (4%)
Recurrence with incomplete records of retreatment	7 (27%)
Incomplete records of recurrence/retreatment	3 (12%)
No recurrence	6 (23%)
Pathologic	
Responder	3 (13%)
Nonresponder	21 (87%)

CTx = chemotherapy; CRTx = chemoradiation therapy; RTx = radiation therapy.

Data are number followed by percentage in parentheses, except for age.

Follow-up of patients previously enrolled in IRB trial 07-03-110 was approved by the UCLA IRB, and the necessity for outcome-specific consent was waived by the IRB for the current trial (IRB study 20-001899).

### ^18^F-FLT PET/CT Imaging and Analysis

Of the 46 ^18^F-FLT PET/CT scans, 43 (93%) were performed on a Siemens Biograph 64 TruePoint PET/CT scanner and 3 (7%) on a Siemens Emotion Duo PET/CT scanner approximately 1 h after a median injected activity of 247.9 MBq (IQR, 229.4–255.3 MBq). Intravenous and oral contrast media were administered in 33 scans (72%) and 36 scans (78%), respectively.

Several SUV parameters were assessed on PET 1 and PET2: SUV_max_, SUV_peak_, SUV_mean_, and SUV for total-lesion FLT with a 40%, 50%, 60%, and 80% cutoff of SUV_max_. Because SUV_max_ proved to be equal or superior to the other PET parameters, we selected SUV_max_ for further analyses. ^18^F-FLT PET/CT images were interpreted by 1 reader. The reader was aware of the sarcoma diagnosis but not of the treatment regimen or other clinical and outcome data.

Posttreatment pathology specimens were assessed by tumor necrosis or fibrosis and by Ki-67 and TK1 expression as described previously (*8*).

### Treatment

Twenty-three of 26 patients (88%) underwent NAT followed by complete surgical resection. Ten patients (38%) underwent neoadjuvant ifosfamide-based treatments, 5 patients (19%) had gemcitabine-based therapy, 1 patient (4%) underwent treatment with doxorubicin (75 mg/m^2^), 1 patient (4%) was treated with paclitaxel (175 mg/m^2^) and bevacizumab, and 1 patient (4%) was treated with ridaforolimus as part of a phase II clinical trial. Standard chemotherapy administrations were previously reported (*8*). Gastrointestinal stromal tumors (*n* = 3; 12%) were treated with imatinib at a dose of 400 mg orally per day. Two patients (8%) received neoadjuvant external-beam radiation only. Ten patients (38%) underwent neoadjuvant chemoradiation therapy. Adjuvant and recurrent treatment regimens are listed in Table 2.

### Histopathology

Pathology specimens were reviewed by a pathologist with expertise in sarcoma pathology, as reported previously (*8*).

### Statistics

Quantitative variables are presented as median and IQR or as mean and SD when appropriate. Statistics were performed using R, version 4.0.2 (R Core Team 2020). SUV cutoffs were delineated using maximally selected rank statistics as implemented in the maxstat R package (http://cran.r-project.org/web/packages/maxstat/index.html). Maximally selected rank statistics evaluated the log-rank comparisons of survival along the continuous absolute SUV_max_ spectrum. Selected cutoffs represent the defined highest threshold for statistical discrimination between values along the SUV_max_ spectrum. Dichotomization via median SUV_max_ was not included because the maximally selected SUV_max_ of 8.5 was equivalent to the median SUV_max_ of 8.7 with low (*n* = 7) and high (*n* = 10) groups for both. Changes in SUV_max_ between PET1 and PET2 were dichotomized at a threshold of 60%. Post-NAT tumor necrosis, Ki-67, and TK1 expression were dichotomized at thresholds of at least 95%, 50%, and 18%, respectively. Survival was calculated from the date of subject consent to the date of death or last follow-up. Deaths included in the survival analysis were categorized as disease-specific death or all-cause mortality, which entailed non–disease-specific death and unknown causes of death. Survival was estimated using the method of Kaplan and Meier. A *P* value less than 0.05 was considered to indicate statistical significance.

## RESULTS

### Outcome Assessment

The cutoff for last follow-up was January 21, 2021. The median follow-up was 104 mo (maximum, 144.8 mo). The median overall survival was 106 mo (95% CI, 31.9–not yet reached [NYR]).

Eleven patients (42%) had no evidence of disease, 10 patients (38%) died of disease, 2 (8%) were alive with disease, and 3 (12%) died of another cause. The median follow-up in patients alive at the last follow-up date was 104 mo (IQR, 27.8–141.1 mo).

### Imaging Characteristics

**FIGURE 1.**
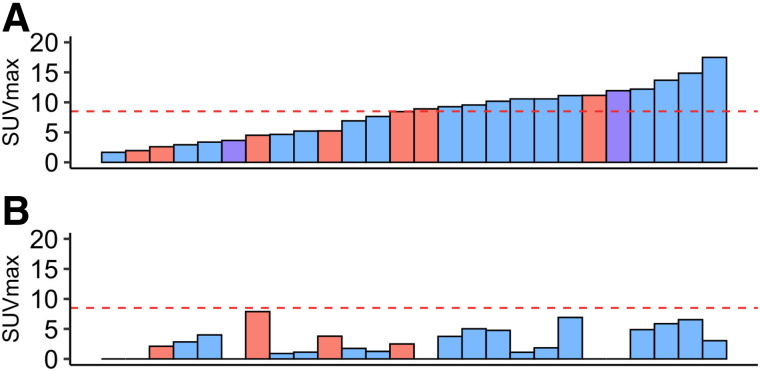
Waterfall diagram of SUV_max_ at PET1 (A) and PET2 (B). Primary tumors are depicted in blue, recurrent or residual tumors in red, and patients with history of secondary malignancy in purple. Red line indicates maximally selected SUV_max_ cutoff of 8.5 to dichotomize patients into low and high baseline ^18^F-FLT uptake.

The tumor SUV_max_ of all patients averaged 6.6 ± 3.7 (median, 7.1; range, 1.7–16.1) and 3.6 ± 2.1 (median, 3.4; range, 0.9–7.9) at PET1 and PET2, respectively (Fig. 1).

The tumor SUV_max_ of primary STS averaged 8.1 ± 4.3 (median, 8.7; range, 1.7–17.5) and 2.8 ± 2.4 (median, 2.3; range, 0–6.9) at PET1 and PET2, respectively.

The tumor size of all patients averaged 8 ± 5 cm (median, 6.4 cm; range, 1.2–20.6 cm) at baseline and decreased to 6.9 ± 3.4 cm (median, 6.1 cm; range, 1.7–14.5 cm) at PET2.

### Imaging Biomarkers

#### PET1

**FIGURE 2.**
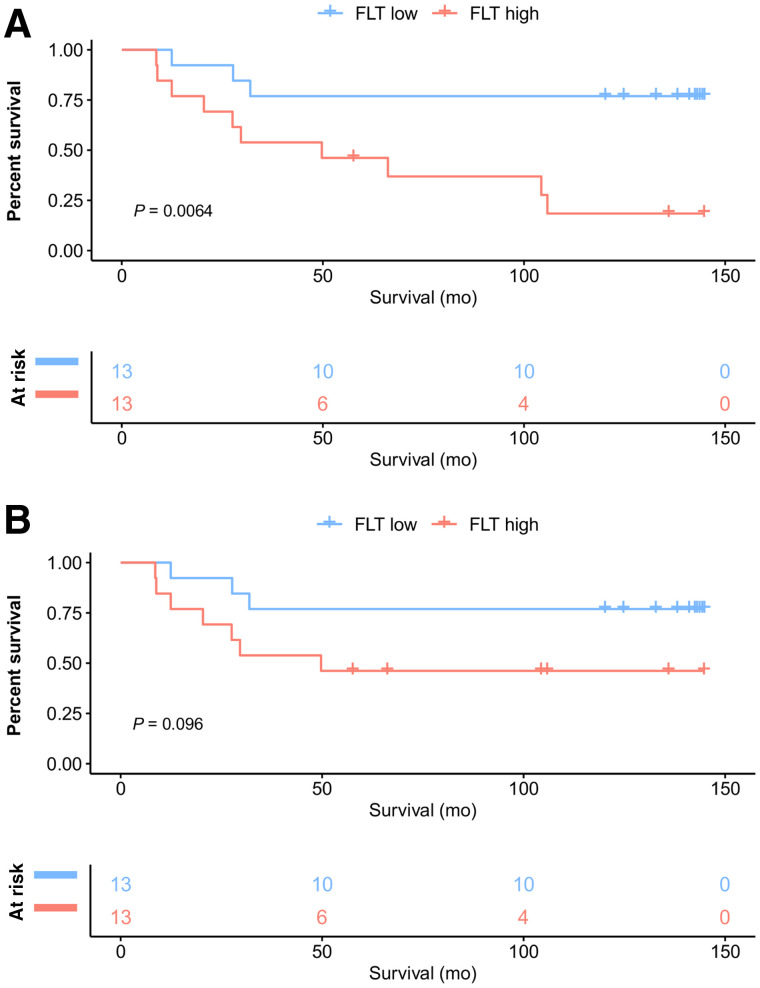
Kaplan–Meier curves for overall survival (A) and DSS (B) in all patients (*n* = 26) dichotomized by SUV_max_ of ≥8.5 vs. <8.5 at PET1.

Overall survival was significantly longer in patients with a low than a high tumor SUV_max_ (dichotomized by an SUV_max_ of ≥8.5 vs. <8.5: NYR vs. 49.7 mo; *P* = 0.0064) (Fig. 2A). DSS showed a trend toward significance (NYR vs. 49.7 mo; *P* = 0.096) (Fig. 2B).

**FIGURE 3.**
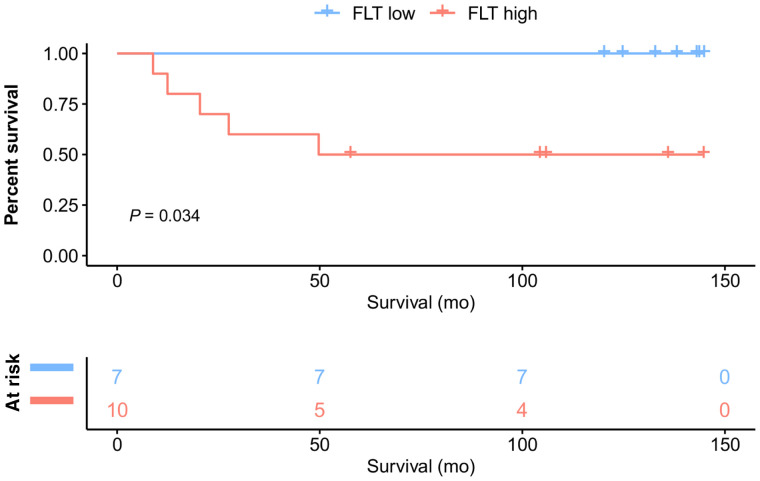
Kaplan–Meier curves for DSS in primary STS (17/26 patients) dichotomized by SUV_max_ of ≥8.5 vs. <8.5 at PET1.

In a subanalysis of primary STS (17/26 patients), DSS was significantly longer in patients with a low than a high tumor SUV_max_ (dichotomized by an SUV_max_ of ≥8 vs <8: NYR vs. NYR; *P* = 0.034) (Fig. 3).

#### PET2 and Changes Between PET1 and PET2

In primary-STS patients who underwent PET2 (*n* = 16/17), neither absolute PET2 tumor SUV_max_ (dichotomized by an SUV_max_ of ≥5 vs. <5: NYR vs. 20.4 mo; *P* = 0.25) nor decreases in SUV_max_ of at least 60% between PET1 and PET2 (NYR vs. NYR; *P* = 0.56) were significantly correlated with DSS survival.

### Histopathologic Biomarkers

DSS in primary-STS patients (16/26 patients) with a histopathologic response in the resected specimens after NAT (*n* = 3) did not significantly differ from that in patients without a post-NAT histopathologic response (*n* = 13) (dichotomized by tumor necrosis and fibrosis of ≥95% vs. <95%: NYR vs. NYR; *P* = 0.86).

Ki-67 expression was available in 14 of 17 primary-STS patients. DSS showed a trend toward being prolonged in patients with a low (*n* = 11) versus a high post-NAT Ki-67 expression (*n* = 3) (dichotomized by an Ki-67 of ≥50% vs. <50%: 27.5 mo vs. NYR; *P* = 0.057) (Supplemental Fig. 1; supplemental materials are available at http://jnm.snmjournals.org).

TK1 expression was available in 14 of 17 primary-STS patients. DSS did not significantly differ between patients with a low post-NAT TK1 expression (*n* = 4) and those with a high post-NAT TK1 expression (*n* = 10) (dichotomized by an TK1 of ≥18% vs. <18%: NYR vs. NYR; *P* = 0.25).

## DISCUSSION

In this post hoc analysis of patients with STS, low pretreatment ^18^F-FLT uptake served as an early prognostic imaging biomarker of long-term survival. The prognostic value of ^18^F-FLT uptake at initial diagnosis has been reported for several malignancies, such as lymphoma (*10*), non–small cell lung cancer (*11*), and pancreatic cancer (*12*). Here, we report the first—to our knowledge—long-term outcomes predicted by baseline ^18^F-FLT uptake in STS patients who underwent NAT.

Because ^18^F-FLT uptake in other tumors has frequently been associated with the proliferation rate of cancer cells, therapy-induced alterations in intratumoral ^18^F-FLT uptake have been proposed as an early imaging biomarker for therapy response and outcome (*6*–*8*). However, in this study ^18^F-FLT uptake after NAT and changes in ^18^F-FLT uptake across treatment did not significantly correlate with improved survival.

Recent literature surrounding the application of ^18^F-FLT illustrates that ^18^F-FLT accumulation is not solely a correlate of tumor cell proliferation rate (*13**,**14*). ^18^F-FLT is a substrate for TK1, a proximal mediator of the pyrimidine salvage pathway that functions in parallel with the de novo pathway to produce deoxythymidine triphosphate for DNA replication and repair (*15*). Thus, ^18^F-FLT avidity is influenced by the relative activity of de novo and salvage pathways, which are in turn regulated by substrate abundance, gene expression, and oncogene or tumor suppressor activity (*15**,**16*). Uptake of ^18^F-FLT is not solely isolated to tumor cells and is impacted by the active proliferation of T cells after removal of cytotoxic T-lymphocyte–associated antigen 4 checkpoint inhibition (*17*). More recently, ^18^F-FLT uptake in tumors has been shown to be elevated alongside interferon signaling–driven thymidine phosphorylase expression in preclinical xenograft models (*18**,**19*). Given that innate and adaptive immune cells are a dominant source of interferon, ^18^F-FLT uptake could also reflect intratumoral immune cell infiltration and elevated cytokine signaling.

An additional potential reason for discordant ^18^F-FLT PET findings after NAT in the current study might be the late timing of PET2 12 wk after the start of NAT. The low ^18^F-FLT uptake at PET2 might in part not represent a cytotoxic treatment effect but viable tumor with low ^18^F-FLT uptake due to restricted tracer delivery, internalization, and trapping. All considered, future studies investigating ^18^F-FLT should integrate clinical observations with a detailed molecular and cellular assessment of biopsy tissue, which could enable the identification of molecular mechanisms driving PET probe accumulation.

Several potential limitations of our study merit consideration. First, this was a small pilot study; therefore, it was not adequately powered to detect small differences. For example, patients with a low post-NAT Ki-67 (≤50%) and a low post-NAT TK1 (≤18%) showed a trend toward a prolonged DSS, but the significance of this finding needs further evaluation. Second, imaging cutoffs were not predefined but maximally selected. Third, patients with a variety of sarcoma subtypes were included in this study.

## CONCLUSION

The current study demonstrates that low ^18^F-FLT uptake at initial diagnosis correlates with long-term survival in primary STS and may be useful in determining treatment strategies. ^18^F-FLT uptake at post-NAT PET does not improve outcome prediction.

## Notes

KEY POINTS **QUESTION:** Can ^18^F-FLT PET/CT be used as a prognostic imaging biomarker of DSS in patients with STS? **PERTINENT FINDINGS:** We report the first long-term outcomes (median follow-up, 104 mo) predicted by ^18^F-FLT PET/CT before initiation of NAT in patients with STS. **IMPLICATIONS FOR PATIENT CARE:** Pretreatment tumor grading guides clinical decision making and prognostication of STS patients. However, given that standard histopathologic grading of STSs has its limitations, new biomarkers are needed to improve clinical management and prognostication and to serve as predictive factors for treatment response.

## References

[1] Siegel RL Miller KD Jemal A. Cancer statistics, 2020. CA Cancer J Clin. 2020;70:7–30. 31912902 10.3322/caac.21590

[2] Casali PG Abecassis N Aro HT. Soft tissue and visceral sarcomas: ESMO-EURACAN Clinical Practice Guidelines for diagnosis, treatment and follow-up. Ann Oncol. 2018;29:iv51–iv67. 29846498 10.1093/annonc/mdy096

[3] Trojani M Contesso G Coindre JM. Soft-tissue sarcomas of adults; study of pathological prognostic variables and definition of a histopathological grading system. Int J Cancer. 1984;33:37–42. 6693192 10.1002/ijc.2910330108

[4] Coindre JM Trojani M Contesso G. Reproducibility of a histopathologic grading system for adult soft tissue sarcoma. Cancer. 1986;58:306–309. 3719523 10.1002/1097-0142(19860715)58:2<306::aid-cncr2820580216>3.0.co;2-7

[5] Lin X Davion S Bertsch EC Omar I Nayar R Laskin WB. Federation Nationale des Centers de Lutte Contre le Cancer grading of soft tissue sarcomas on needle core biopsies using surrogate markers. Hum Pathol. 2016;56:147–154. 27346575 10.1016/j.humpath.2016.06.008

[6] Amin MB Edge S Greene F, eds. AJCC Cancer Staging Manual. 8th ed. Springer International Publishing; 2017.

[7] Chibon F Lagarde P Salas S. Validated prediction of clinical outcome in sarcomas and multiple types of cancer on the basis of a gene expression signature related to genome complexity. Nat Med. 2010;16:781–787. 20581836 10.1038/nm.2174

[8] Benz MR Czernin J Allen-Auerbach MS. 3′-deoxy-3′-[^18^F]fluorothymidine positron emission tomography for response assessment in soft tissue sarcoma: a pilot study to correlate imaging findings with tissue thymidine kinase 1 and Ki-67 activity and histopathologic response. Cancer. 2012;118:3135–3144. 22020872 10.1002/cncr.26630 PMC3436595

[9] Yap CS Czernin J Fishbein MC. Evaluation of thoracic tumors with ^18^F-fluorothymidine and ^18^F-fluorodeoxyglucose-positron emission tomography. Chest. 2006;129:393–401. 16478857 10.1378/chest.129.2.393

[10] Herrmann K Buck AK Schuster T. Predictive value of initial ^18^F-FLT uptake in patients with aggressive non-Hodgkin lymphoma receiving R-CHOP treatment. J Nucl Med. 2011;52:690–696. 21498532 10.2967/jnumed.110.084566

[11] Scheffler M Zander T Nogova L. Prognostic impact of [^18^F]fluorothymidine and [^18^F]fluoro-D-glucose baseline uptakes in patients with lung cancer treated first-line with erlotinib. PLoS One. 2013;8:e53081. 23308140 10.1371/journal.pone.0053081 PMC3537767

[12] Wieder H Beer AJ Siveke J. ^18^F-fluorothymidine PET for predicting survival in patients with resectable pancreatic cancer. Oncotarget. 2018;9:10128–10134. 29515797 10.18632/oncotarget.24176 PMC5839378

[13] Shields AF. PET imaging of tumor growth: not as easy as it looks. Clin Cancer Res. 2012;18:1189–1191. 22275505 10.1158/1078-0432.CCR-11-3198 PMC3294137

[14] Zhang CC Yan Z Li W. [^18^F]FLT-PET imaging does not always “light up” proliferating tumor cells. Clin Cancer Res. 2012;18:1303–1312. 22170262 10.1158/1078-0432.CCR-11-1433

[15] Nathanson DA Armijo AL Tom M. Co-targeting of convergent nucleotide biosynthetic pathways for leukemia eradication. J Exp Med. 2014;211:473–486. 24567448 10.1084/jem.20131738 PMC3949575

[16] Villa E Ali ES Sahu U Ben-Sahra I. Cancer cells tune the signaling pathways to empower de novo synthesis of nucleotides. Cancers (Basel). 2019;11:688. 10.3390/cancers11050688 PMC6562601 31108873

[17] Ribas A Benz MR Allen-Auerbach MS. Imaging of CTLA4 blockade-induced cell replication with ^18^F-FLT PET in patients with advanced melanoma treated with tremelimumab. J Nucl Med. 2010;51:340–346. 20150263 10.2967/jnumed.109.070946

[18] Schelhaas S Heinzmann K Honess DJ. 3′-deoxy-3′-[^18^F]fluorothymidine uptake is related to thymidine phosphorylase expression in various experimental tumor models. Mol Imaging Biol. 2018;20:194–199. 28971330 10.1007/s11307-017-1125-3

[19] Eda H Fujimoto K Watanabe S. Cytokines induce thymidine phosphorylase expression in tumor cells and make them more susceptible to 5′-deoxy-5-fluorouridine. Cancer Chemother Pharmacol. 1993;32:333–338. 8339382 10.1007/BF00735915

